# Anisotropic Finite Element Modeling Based on a Harmonic Field for Patient-Specific Sclera

**DOI:** 10.1155/2017/6073059

**Published:** 2017-02-07

**Authors:** Xu Jia, Shenghui Liao, Xuanchu Duan, Wanqiu Zheng, Beiji Zou

**Affiliations:** ^1^Department of Ophthalmology, The Second Xiangya Hospital, Central South University, Changsha, Hunan 410011, China; ^2^School of Information Science and Engineering, Central South University, Changsha, Hunan 410083, China

## Abstract

*Purpose*. This study examined the influence of anisotropic material for human sclera.* Method*. First, the individual geometry of patient-specific sclera was reproduced from a laser scan. Then, high quality finite element modeling of individual sclera was performed using a convenient automatic hexahedral mesh generator based on harmonic field and integrated with anisotropic material assignment function. Finally, comparison experiments were designed to investigate the effects of anisotropy on finite element modeling of sclera biomechanics.* Results*. The experimental results show that the presented approach can generate high quality anisotropic hexahedral mesh for patient-specific sclera.* Conclusion*. The anisotropy shows significant differences for stresses and strain distribution and careful consideration should be given to its use in biomechanical FE studies.

## 1. Introduction

Recently, studies have focused on understanding the properties of sclera biomechanics because the biomechanical properties of the sclera and lamina cribrosa determine the biomechanical changes of the optic papilla [1, 2], playing an important role in the process of RGC loss and optic nerve damage caused by increased in intraocular pressure (IOP) [3, 4].

The posterior part of eyeball consists of three layers: the sclera, choroid, and retina. The sclera is the toughest of these three tissues and the retina is the softest. The posterior sclera is divided into two layers where it meets the optic nerve. The outer two-thirds form the scleral canal which transitions to the optic sheath, and the inner third forms the lamina cribrosa, an irregular mesh-like structure through which the optic nerves traverse the eyeball. Subjected to the same stress, the tangent modulus of the retina, choroid, and sclera differs by an order of magnitude, with the sclera being the highest. Thus, the sclera plays a crucial role in maintaining the shape of the eyeball. The sclera is the outer coating of the eye and is comprised of fibrous tissue, consisting almost entirely of dense bands of parallel and interlacing collagen that maintains the biomechanical properties of the sclera [5]. Studies have found that the sclera tissue of most animals is structurally anisotropic with several consistent features. In the posterior and peripapillary region, the scleral fibers were mostly circumferential but less aligned than those in the anterior and equatorial regions [6]. Circumferential scleral fibers may act as reinforcing rings to limit optic nerve head deformations. The biomechanical properties of the scleral collagen fibrils show anisotropic response to external force [7].

As a numerical modeling method for structure analysis suitable for complex material properties and complex boundary conditions of biological structures, finite element (FE) analysis has become a popular analytical tool in biomechanical studies.

Earlier finite element models were simple linear, elastic, homogeneous, and isotropic materials [8]. Pinsky and Datye [9] developed a linear material corneal model based on the anisotropic constitutive model using the predominant fiber directions in the cornea, representing the stroma as a nearly incompressible, isotropic, hyperelastic material. Anisotropic nonlinear hyperelastic models with embedded collagen fibers have been used in recent corneal models [10–14]. Alastrué et al. [10] used a discrete fiber model with embedded collagen fibers in two preferred orientations, the nasal-temporal and superior-inferior directions. Most other recent models do not simulate each fiber explicitly, which can be computationally expensive; however, they include a smeared effect of oriented fibers. Pandolfi and Pinsky et al. developed their models with two preferred directions at the center and circumferential at the limbus region [11–14]. Nguyen et al. [14] created an anisotropic corneal model, taking into consideration the viscoelastic properties. Uchio et al. revealed that the sclera exhibits anisotropic and viscoelastic characteristics and the posterior sclera shows more extensive deformation than the anterior and peripheral sclera under the same stress [15].

Other studies showed that the stress borne by ocular tissues was determined by the 3D geometrical shape of the tissue, and IOP-generated stress and the corresponding response occurred in different regions of the sclera [16], in other words, the similarity of the FE model to the real structure of the analyzed object. Excessive simplifications in geometry will inevitably result in considerable inaccuracy. The geometric modeling, however, is a rather difficult task when automatic mesh generators (AMG) are not available.

There are two main classes of primitives that are used for 3D finite element modeling: tetrahedral and hexahedral elements, each with their own advantages and disadvantages. Tetrahedral elements are geometrically versatile and can be reliably generated by many automatic meshing algorithms, even for complex shape models. However, a good mesh of hexahedral elements can vastly reduce the number of elements and consequently reduce analysis and postprocessing times. In addition, hexahedral elements may be more suited for nonlinear analysis or situations where the alignment of elements is important to the physics of the problem, such as in computational fluid dynamics or the simulation of anisotropic materials. Many hexahedral meshing approaches have been proposed [17]: feature-based, medial surface subdivision, plastering, grid-based, whisker weaving, and others. These traditional methods were mainly designed for regular CAD models but are not well-suited for biomechanical models with irregular and complex shapes. Grosland et al. [18] developed an open-source software toolkit (IA-FEMesh). IA-FEMesh employs a multiblock meshing scheme aimed at hexahedral mesh generation. An emphasis has been placed on making the tools interactive, in an effort to create a user friendly environment. They demonstrated several biomechanical hexahedral meshing models. We noticed that these methods do not consider the anisotropic material of human tissues.

Some studies have employed regular mapping hexahedral meshing to generate regular hemisphere sclera with the same shell thickness [19], a strategy that would not be suitable for the individual geometry of patient-specific sclera. The shape of real sclera is not a regular shape, and there is significant intraregion variation in sclera thickness. Pandolfi et al. [11, 14] developed parameter-based mesh generator to model the patient-specific cornea. Based on a two-dimensional grid generation algorithm, the mesh generator creates the structure of the cornea. The input is limited to a few geometrical parameters, which describe the internal and external surfaces of the cornea in terms of biconic functions. Ariza-Gracia et al. [13] presented automatized patient-specific methodology for numerical determination of biomechanical corneal response. A surface continuation algorithm is proposed to extended corneal surface required in order to achieve a 12 mm diameter, which could introduce some geometric error in the periphery region due to the smoothing algorithm (<1%).

The goal of this study was to investigate a convenient automatic hexahedral mesh generator for patient-specific sclera based on a harmonic field. The basic idea was to smoothly distribute a regular hexahedral mesh on the irregular patient-specific sclera. The harmonic field is one of the most efficient tools for smooth distribution [20]. Its gradient vector field and isocontours field flow smoothly on the surface of model and are perpendicular to each other, which can be used to represent scleral predominant fiber directions, as well as to drive the hexahedral meshing scheme. In addition, the Laplace–Beltrami operator for the surface mesh approximates the normal mean curvature of the model, so that the distribution pattern of harmonic field tends to conform very well to the shape of the model. We used sampled streamlines of the gradient vector field and isocontours of the harmonic field to generate the hexahedral mesh. These sampled streamlines flow exactly on the surface of model, which can preserve the original sclera shape to generate “patient-specific” mesh, so as to effectively handle the regular shape of patient-specific sclera and significant intraregion variation in sclera thickness. An anisotropic material assignment procedure can be easily integrated based on the same harmonic field. We also investigated to what extent anisotropic elastic properties affect the stress and strain distribution of sclera under physiologic loads.

The remaining of this paper is organized as follows. The details of our work are explained in Section 2. In Section 3, the experimental results are reported and discussed. Finally, we present our conclusions in Section 4.

## 2. Methodology

**Figure 1:**
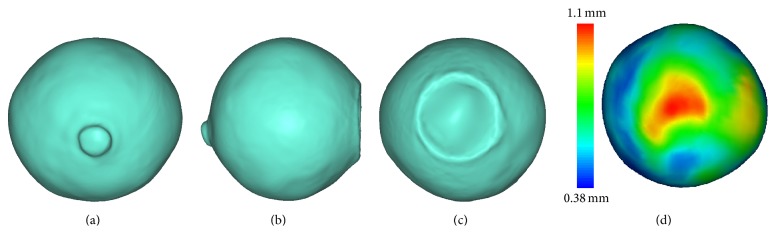
Geometry model of patient-specific sclera. (a)–(c) Posterior, temporal, and front views of 3D scan model. (d) Posterior sclera with thickness contour superimposed.

The starting point of our investigation was a well-reproduced individual geometry of a patient-specific sclera, produced by a high-precision laser scanner as shown in Figures 1(a)–1(c). The sclera solid model consists of an outer surface and an inner surface, represented by triangular piecewise linear surface mesh. As the posterior sclera around the optic nerve head (ONH) is a region of particular interest [21, 22], we extracted the posterior hemisphere model and the thickness contour was computed and superimposed. The thickest region of the sclera was observed at the posterior pole of the eye, 1.1 mm, and the thinnest sclera occurred at the equator, measuring 0.38 mm.

### 2.1. Hexahedral Sclera Meshing Based on Harmonic Field

As demonstrated in Figure 1, significant intraregion variation in sclera thickness was observed: the average ratio between the mean thicknesses of the thickest and thinnest regions of sclera on an eye was about 3 : 1. This variation combined with the irregular shape of the sclera means that regular mapping hexahedral meshing as used in previous studies to generate regular hemisphere sclera with uniform shell thickness is not suitable for the individual geometry of our patient-specific sclera.

Instead, we investigated an ad hoc automatic hexahedral meshing for the posterior sclera, initially without the ONH region for simplicity. The basic idea was to smoothly distribute a regular hexahedral mesh on the irregular patient-specific sclera.

**Figure 2:**
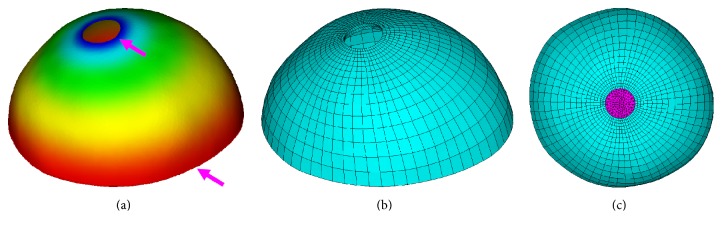
Hexahedral sclera meshing based on harmonic field. (a) Color mapped harmonic field. (b) Hexahedral mesh of posterior sclera. (c) Mesh of posterior sclera and ONH.

One of the most efficient tools for smooth distribution is the harmonic field, which has no local extrema other than at the constrained vertices [20]. If all constrained minima are assigned the same global minimum value and all constrained maxima are assigned the same maximum value, then all the constraints will be guaranteed to be extrema in the resulting field, and the gradient vector field will converge precisely at the specified constraint points and flow smoothly at all other locations. In addition, the Laplace–Beltrami operator for the surface mesh approximates the normal mean curvature, so that the scalar distribution pattern of harmonic field tends to conform very well to the shape of the surface. These characteristics are the very properties we require in our application. In other words, we just need to position the minima and maxima constraints on the end circles around the ONH and the equator, as referred by the purple arrow in Figure 2(a), to generate the harmonic field required to drive the meshing scheme.

In more detail, for both the outer surface and inner surface mesh of posterior sclera, we would like to construct a harmonic field *f* such that Δ*f* = 0, where Δ is the Laplacian operator, subject to the Dirichlet boundary conditions. The standard definition of the Laplacian operator on a piecewise linear surface mesh *M* is the umbrella operator

(1) $$\begin{array}{c} \Delta f_{i}=\overset{}{\underset{j\in N\left(i\right)}{\sum }}w_{ij}\left(\;f_{i}-f_{j}\;\right), \end{array}$$

where *j* ∈ *N*(*i*) is the set of vertices adjacent to vertex *i* and *w*_*ij*_ is a scalar weight assigned to the edge (*i*, *j*). The standard choice for the weights *w*_*ij*_ of surface mesh is the discrete harmonic weights *w*_*ij*_ = (1/2)(cot⁡*α*_*ij*_ + cot⁡*β*_*ij*_), where *α*_*ij*_ and *β*_*ij*_ are the angles opposite the edge. Assembling the vertices' function values *f*_*i*_ into an *n*-vector **f**, the Laplacian operator can be written in matrix form **Lf** = 0, where the elements of the *n* × *n* matrix **L** are given by

(2) $$\begin{array}{c} L_{ij}=\left\{\begin{array}{cc} \overset{}{\underset{k\in N_{1}\left(i\right)}{\sum }}w_{ik}, & \text{if }i=j\\ -w_{ij}, & \text{if }j\in N\left(i\right)\\ 0, & \text{otherwise}. \end{array}\right. \end{array}$$

Eliminating the rows and columns corresponding to the constraint vertices by bringing them to the right-hand side yields a linear system of the form **Ax** = **b**, consisting of the positive definite sparse matrix **A** and a right-hand-side vector **b**. Iterative methods such as the preconditioned conjugate gradient method can efficiently obtain the solution of the linear system.

The resulting harmonic field on the outer surface of sclera is illustrated in Figure 2(a), and the scalar values are mapped to colors (blue represents small values and red represents large values).

For both the outer and inner surface of the posterior sclera, a group of even sampled seed points were placed on the end circles around the equator and were traced by the directions of the gradient vector field derived from the harmonic scalar field. These streamlines of gradient flow smoothly and converge precisely at the end circles around the ONH. At the same time, a group of isocontours for the harmonic scalar field were sampled by even scalar values. These gradient streamlines and the isocontours formed a perfect quadrilateral meshing for the outer and inner surfaces of the posterior sclera. We additionally insert a middle interpolation layer of quadrilateral mesh between the outer and inner surface. These 3 layers of quadrilateral meshes provide the sample topology and could be connected automatically to generate the complete hexahedral mesh of the sclera, as illustrated in Figure 2(b). Finally, these 8-node linear hexahedral elements are converted to 20-node nonlinear hexahedral elements, to account for large material deformation and increase the accuracy of the finite element model.

It should be noted that although the shape of patient-specific posterior sclera is not a regular hemisphere and the ONH is not located in the center pole of the hemisphere, the resulting hexahedral mesh still shows very regular grid cell arrangement. In addition, the mesh element is adaptively distributed with higher density near the ONH region and lower density near the equator.

### 2.2. Anisotropic Material Setting Based on Harmonic Field

According to previous studies [23], the sclera exhibits anisotropic characteristics and scleral fibers are mostly circumferential with respect to the ONH. In the peripapillary scleral region, the fibers are less aligned than those in the anterior and equatorial regions. In other words, we need to define a local material axis coordinate for each element cell in the FEM as part of anisotropic material setting. This step is simple if we were using a regular hemisphere sclera model, but it is more difficult for the irregular patient-specific sclera to generate orthotropic principal axes vector fields that change from point to point inside tissues [24].

**Figure 3:**
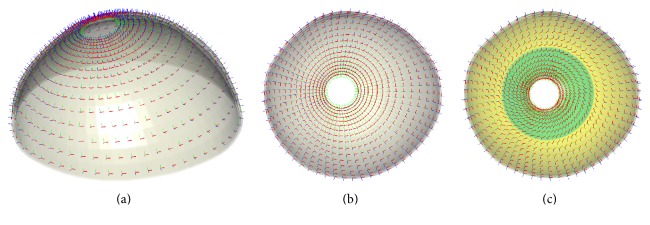
Anisotropic material axis coordinates of the sclera. (a)-(b) Sclera fibers are perfect circumferential. (c) Peripapillary sclera fibers rotating 30° and peripheral fibers rotating 40°.

To address this challenge, we used the already existing harmonic field. It is obvious that the tangent direction of these isocontours is compatible with the trajectory of the circumferential sclera fibers and the direction of the gradient vectors is compatible with the trajectory of the meridional fibers. Specifically, we set a local material coordinate for each element cell in the model, using the circumferential direction as the *x*-axis and the gradient direction as the *y*-axis, such as the red and green axis vectors shown in Figures 3(a) and 3(b). The cross production of the circumferential and gradient direction generated the *z*-axis, indicating the sclera thickness direction.

For the anisotropic elastic material constants of the sclera, we set the *E*_*x*_ = 8.6 MPa, *E*_*y*_ = 6 MPa, and *E*_*z*_ = 2.5 MPa, based on previous studies [25, 26]. Tissues were assumed to be incompressible, although a Poisson ratio of 0.49 rather than 0.50 was selected to avoid nonconvergent numerical behavior.

To determine to what extent anisotropic characteristics affect the stress and strain distribution of sclera, we also built an isotropic sclera FE model and calculated the effective isotropic properties to take the spatial average of the elastic constants in all directions. This was accomplished using two averaging methods that provided upper and lower bounds for the transformed isotropic constants. We averaged the upper and lower bounds to obtain the constants using the isotropic elastic *E* = 3.8 MPa, compatible with previous studies [26]. In addition, as the previous studies pointed out, the scleral fibers are mostly circumferential with respect to the ONH in the peripapillary scleral region, but less aligned than those in the anterior and equatorial regions; we built a group of comparison models based on the ideal anisotropic sclera model with perfect circumferential fibers [6, 27]. We first rotated the fibers in the peripheral region by 10° in the “r1” model, rotated the fibers in the peripapillary region by 10° and the peripheral region by 20° in the “r2” model, rotated the fibers in the peripapillary region by 20° and in the peripheral region by 30° in the “r3” model, and rotated the fibers in the peripapillary region by 80° and in the peripheral region by 90° in the “r10” model. The r4 model is illustrated in Figure 3(c).

For each model, the boundary conditions constrained all three degrees of freedom at each of the nodes located at the scleral equator. Loading was simulated by applying an intraocular pressure of 30 mmHg on the inner surface of the posterior sclera.

## 3. Results

### 3.1. Hexahedral Mesh Generator for Sclera

For the patient-specific sclera model in Figure 2, our hexahedral sclera mesh generator based on harmonic field produced 5410 hexahedral elements. Note that 20-node nonlinear hexahedral elements are used to account for large material deformation and increase the accuracy of the finite element model. The total computation time required 938 ms. To test the quality of the mesh used for the calculations, a sensitivity analysis was performed. The results show that the changes of maximal displacement are less than a 0.1%, whereas for the maximum principal stress is less than 2.5%, demonstrating the adequacy of the used mesh.

**Figure 4:**
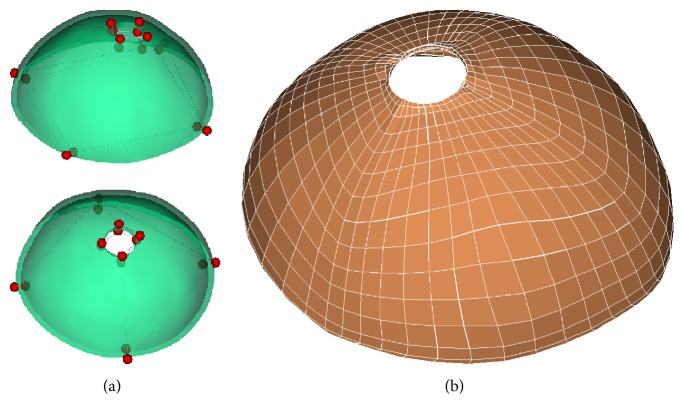
Hexahedral sclera meshing by IA-FEMesh. (a) Manual edit of multiblock structure for meshing. (b) Hexahedral mesh of posterior sclera.

As discussed before, some studies employed regular mapping hexahedral meshing to generate regular hemisphere sclera with uniform shell thickness [19], which is not suitable for the individual geometry of our patient-specific sclera because real sclera is not shaped like a regular sphere and there is significant intraregion variation in sclera thickness. For comparison to our model, we used the IA-FEMesh generator which employs a multiblock meshing scheme aimed at hexahedral mesh generation [18]. Where nontrivial boundaries are required, “block-structured” techniques are employed to allow the user to manually break the domain into topological blocks, as shown in Figure 4(a). This block generation procedure took us about 3 minutes. The computational procedure includes mainly two steps. Firstly, closest-point projection is used to morph the surface nodes of the rectilinear unstructured mesh onto the underlying surface of interest. After the surface nodes are established, the user can use either elliptical or transfinite interpolation to compute the interior nodes. Using the same input sclera model, the total mapping computation time required about 2 minutes.

Statistical analysis of the dihedral angle distortion from perfect hexahedral element shows that our approach produced more high quality mesh elements, as demonstrated in Table 1, where we can observe that about 85% of the dihedral angle distortions are less than 20° in our method, but there are only about 50% in the IA-FEMesh generator. The average dihedral angle distortions of the two methods are 7.8° and 17.5°, respectively, and the maximum angle distortions are 43° and 86°. In other words, the IA-FEMesh produced flipped or self-intersected elements, as Grosland et al. reported, requiring a postsmoothing procedure to detect irregular elements and improve element quality [18].

In the IA-FEMesh generator, the closest-point projection algorithm results in some mesh vertices that deviate from the input model, especially on the sharp feature edges. In contrast, our harmonic mesh generator requires that all mesh vertices are precisely on the input model and thus better preserves the original geometric shape of the patient-specific sclera model.

In addition, an effective anisotropic material assignment procedure can be easily integrated with our mesh generator based on the same harmonic field. The IA-FEMesh generator does not allow consideration of anisotropic material.

### 3.2. FEM Simulation Analysis for Sclera

**Figure 5:**
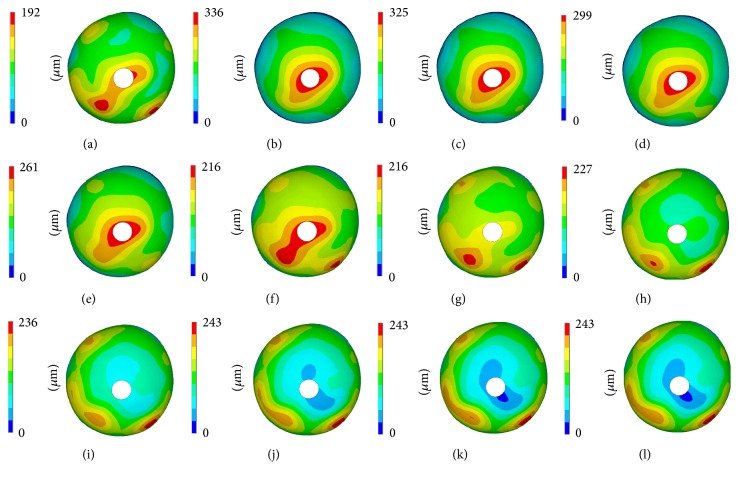
Displacement contour of the sclera. (a) Isotropic model. (b) Perfect anisotropic model. (c)–(l) R1 model to R10 model.

**Figure 6:**
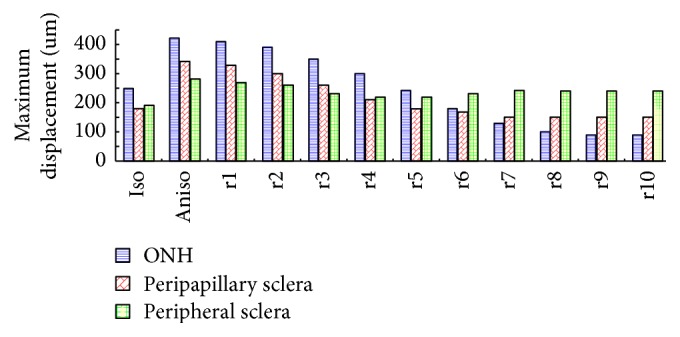
Maximum displacement value of the ONH region, peripapillary and peripheral sclera.

Figures 5 and 6 illustrate the displacement contour of the sclera as well as the maximum displacement value of the ONH region, peripapillary, and peripheral sclera. We can see that the isotropic model with less displacement is harder than the anisotropic model, and the maximum displacement value occurred around the ONH region for the perfect anisotropic model, consistent with previous studies [15]. When the rotation angle of the scleral fibers increased, the displacement of the ONH and peripapillary sclera decreased and the displacement of peripheral sclera increased.

**Figure 7:**
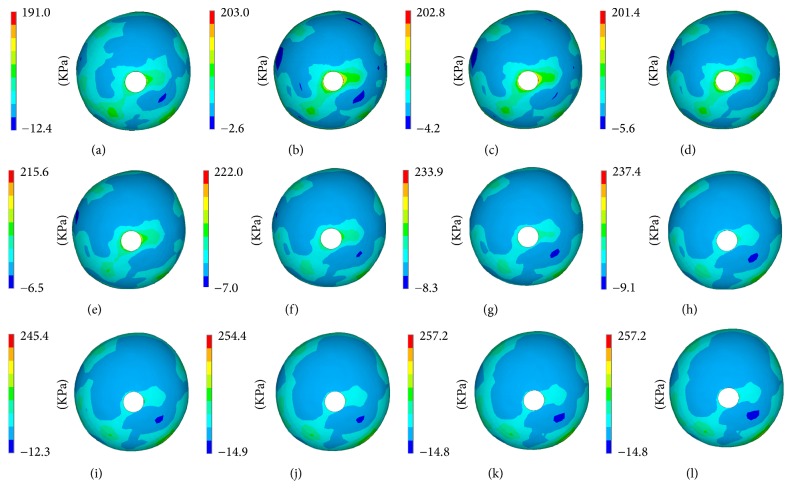
Maximum principal stress of the sclera. (a) Isotropic model. (b) Perfect anisotropic model. (c)–(l) R1 model to R10 model.

**Figure 8:**
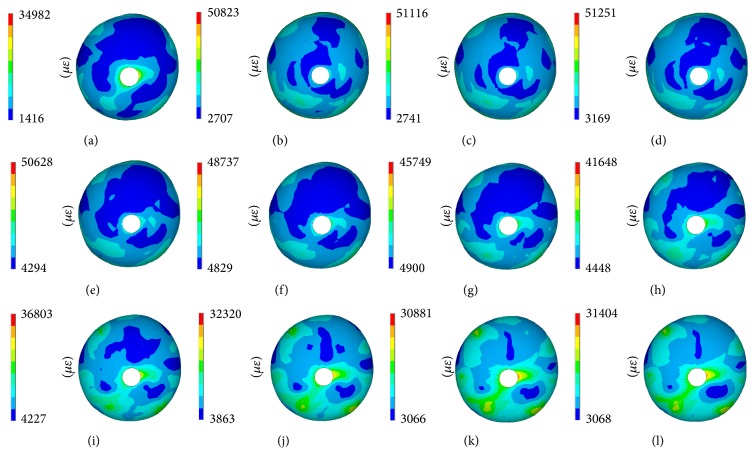
Maximum principal strain of the sclera. (a) Isotropic model. (b) Perfect anisotropic model. (c)–(l) R1 model to R10 model.

**Figure 9:**
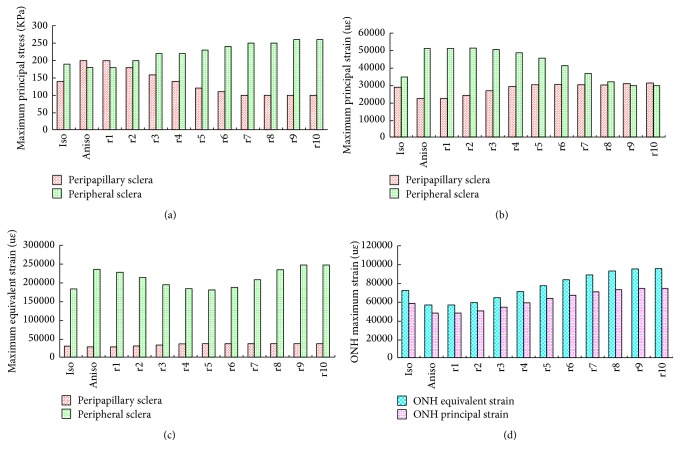
Maximum stress and strain value. (a) Maximum principal stress of the sclera. (b) Maximum principal strain of the sclera. (c) Maximum equivalent strain of the sclera. (d) Maximum equivalent and principal strain of the ONH.

Figures 7 and 8 show the maximum principal stress and strain contour of the sclera, and Figure 9 illustrates the maximum stress and strain values. We can see that the principal stress in the isotropic model is less than that of the anisotropic model, and the maximum stress value occurred around the ONH region for the perfect anisotropic model, as shown in previous studies [25]. When the rotation angle of the scleral fibers from the ideal circumferential direction increased, the stress of the peripapillary sclera decreased, and the stress of peripheral sclera increased. On the other hand, the principal strain in the isotropic model was larger than the anisotropic model, and when the rotation angle of the scleral fibers from the ideal circumferential direction increased, the stress of the peripapillary sclera increased, and the stress of peripheral sclera decreased. The strain in the ONH region was similar to the peripapillary sclera; that is, the strain in the isotropic model is larger than the anisotropic model, and the strain increased when the rotation angle of the scleral fibers from idea circumferential direction increased.

## 4. Conclusions and Discussion

The important pathological feature of glaucoma is the atrophy of the optic nerve and the thinning of retinal nerve fiber layer. The maximum change of stress occurred in the posterior part of the eyeball around ONH, which means that the posterior part of sclera and ONH suffer the most stress. From a biomechanical perspective, the eyeball is a pressure vessel. IOP can produce tissue deformation, strain, and stress. ONH is closely related to visual function, but it is difficult to detect the effect of IOP on the scleral lamina cribrosa due to complicated effects of extension, compression, and shearing [28]. The sclera is the main force ocular tissue and the deformation of the scleral tissue can be transferred to the ONH by the scleral canal. So, the biomechanical characteristics and behavior of the sclera may affect the response of ONH to IOP changes.

The sclera is considered rigid material with high stress with low strain. The scleral tissue is complex and exhibits anisotropy [29], nonlinearity [30], and viscoelasticity [31]. Nonlinearity is a characteristic of most soft tissue, often due to the presence of collagen fibers in the tissue. When the pressure vessel of the nonlinear material is deformed, softening and stiffness exist simultaneously. The stiffness of the sclera will increase dramatically when the intraocular pressure increases dramatically [19]. Anisotropic materials exhibit different values for a given property in different directions. The trend and distribution of collagen fibers determine the deformation of the sclera, but the exact shape of the collagen fibers of sclera is not clear [32].

This paper presents a novel method to generate a patient-specific hexahedral mesh of a sclera model based on harmonic field that can effectively handle the regular shape of patient-specific sclera and the significant intraregion variation in sclera thickness. The experimental results demonstrated that the method is computationally efficient. This approach can generate high quality hexahedral meshes for patient-specific sclera models and avoids element flipping and mesh angle distortions to a large extent, eliminating the need for further postsmoothing procedures to detect irregular elements and improve element quality. In addition, an anisotropic material assignment procedure can be easily integrated based on the same harmonic field.

Experimental results showed that the maximum displacement of scleral tissue closest to the ONH is larger than the tissue farther away from the ONH. Additionally, the scleral tissue around the ONH is the most concentrated area of stress as intraocular pressure increases. These results are similar to previous findings in animal experiments [25]. The biomechanical response of sclera to elevated IOP is predominantly centered in the tissue immediately surrounding the ONH. Different creep mechanisms may be activated at different stress levels generated by IOP [14]. When IOP initially increases, the displacement caused by the increased stress may be regulated by the collagen fibers. As IOP continues to increase, further displacement may be caused by stretching of the collagen fibrils. At the same time, the maximum strain and displacement occur in the peripapillary sclera, which means that the peripapillary sclera and ONH exhibit the greatest morphological changes. It was previously thought that the elevated IOP pushed the lamina cribrosa posteriorly, without causing a significant degree of scleral deformation [33]. However, the most recent data from numerical and experimental studies show that when IOP increases, the sclera deforms, often influencing the displacement of the lamina cribrosa by transmitted deformations to the ONH [34]. The results suggest that to diagnose early glaucoma or judge the severity and type clearly, we should understand the morphological changes of optic papilla and peripapillary sclera using Optical Coherence Tomography as well as the ophthalmoscope.

## Figures and Tables

**Table 1:** Statistics analysis on the element dihedral angle distortion.

	Our method	Mesh matching method
0°*～*10°	41.2%	19.7%
10°*～*20°	43.6%	30.2%
20°*～*30°	11.3%	20.3%
30°*～*40°	2.3%	14.1%
40°*～*50°	1.6%	9.3%
>50°	0%	6.4%
Maximum	43°	86°
Average	7.8°	17.5°

## Additional Points

Finite element modeling is an efficient way to predict biological tissue behavior under physiologic loads. Various experiments have sought to identify the factor governing the ability of a given sclera and ONH to maintain structural integrity [26], nutritional homeostasis, and axoplasmic transport at physiological and pathological IOP levels [35]. In engineering, there are still many remaining questions about the mechanism and clinical recognition of glaucoma, including the pathogenesis and distribution of IOP-related nerve damage in the peripheral scleral around ONH. In the future, we will continue to develop and improve the finite element model of human sclera to allow more accurate simulation to continue to investigate these mechanisms. A preferred direction of research is to expand our modeling method with the anisotropic nonlinear hyperelastic models with embedded collagen fibers [10–14].
